# Supplementary figures and images for: Transcriptomic analysis of N-terminal mutated Trypanosoma cruzi UBP1 knockdown underlines the importance of this RNA-binding protein in parasite development
Source: PLoS Negl Trop Dis. 2024 May 17;18(5):e0012179. doi: 10.1371/journal.pntd.0012179 (PMC11139272; doi:10.1371/journal.pntd.0012179)

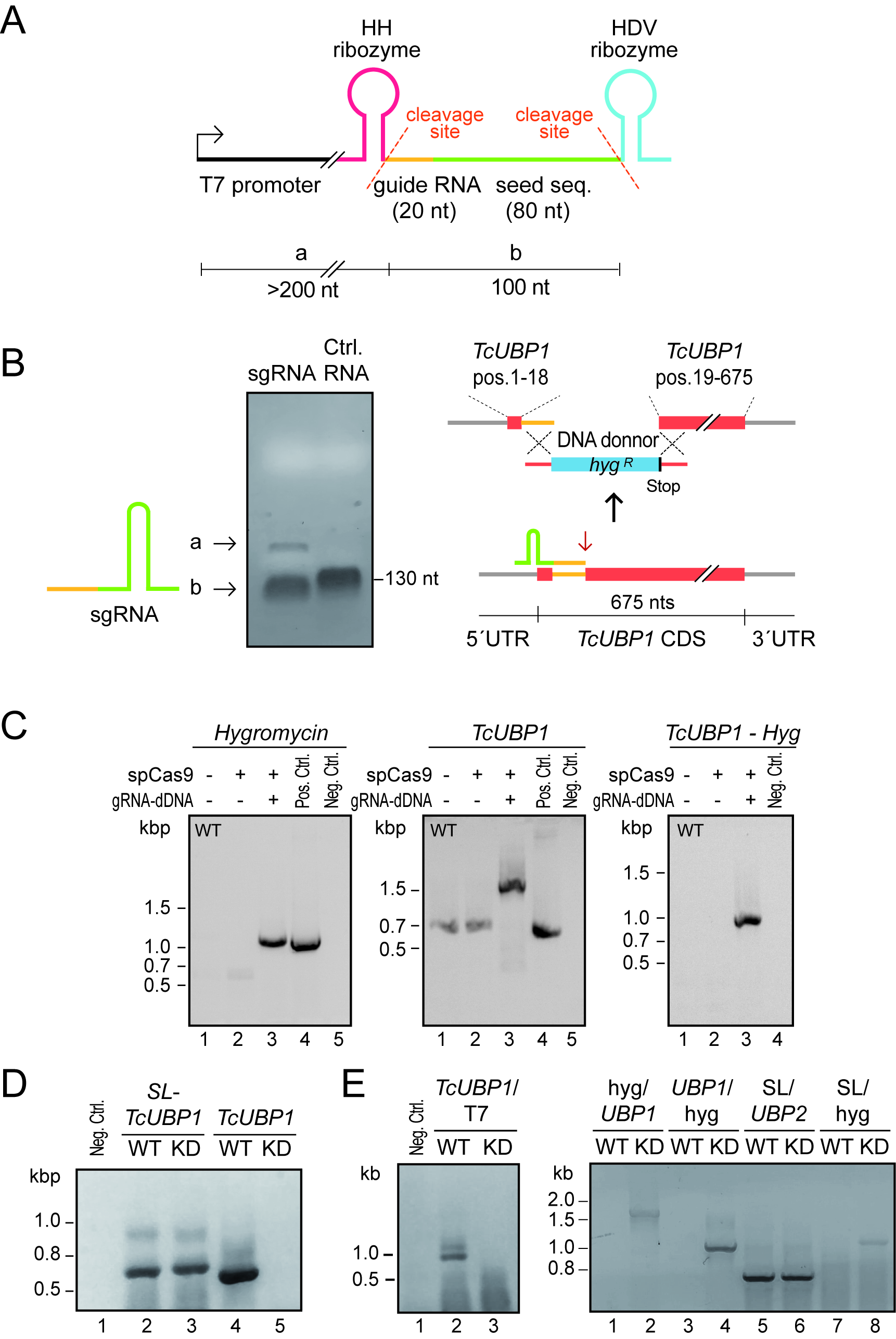

Supplement: S1 Fig — A, Scheme of the gRNA construct cloned into pEASY-T1 used for in vitro transcription. The T7 promoter region (black), HH ribozyme (magenta), the gRNA target complementary sequence (orange), the gRNA target sequence (green), and the cleavage sites of the ribosomes (dotted lines) are marked. B, Ethidium bromide-stained 2% agarose gel with denatured samples of the in vitro transcription gRNA product and a known-length RNA marker used as size control (Ctrl. RNA, 130 nt). The band of greater size corresponds to the gRNA upstream sequence (a), and one smaller band that is the gRNA sequence (b) are indicated with arrows; the sequence downstream to the gRNA is not observed in the gel due to its low size (left), Scheme of the construct obtained after transfection with the complete system. The hygromycin resistance CDS was used as donor DNA (cyan) flanked by homologous sequences on both sides to the spCas9 site (tomato). The spCas9 cutting site is indicated within the coding sequence of TcUBP1 (red arrow), TcUBP1 CDS (tomato), 5’ and 3′-untranslated region of TcUBP1, and target sequence recognized by the gRNA (orange) (right). C, Agarose gel electrophoresis of PCR products from genomic DNA extracted from wildtype (WT) populations (lane 1), spCas9-GFP (lane 2) or populations transfected with the complete system (spCas9-gRNA-DNA donor) (lane 3) generated using forward and reverse-specific primers for hygromycin, for TcUBP1, or a combination of specific forward oligo for TcUBP1 and reverse oligo for hygromycin. D, PCR products from cDNA of KD or WT parasites generated using a forward primer specific to the Spliced leader sequence and a reverse primer specific to the TcUBP1 CDS; or with both specific primers for TcUBP1 CDS. E, PCR products from cDNA of KD or WT parasites generated using a combination of forward/reverse primers specific for TcUBP1, TcUBP2, SL, T7 or hygromycin. Molecular mass protein standards or DNA markers are indicated on the left. Pos. Ctrl., positive PCR cont [file pntd.0012179.s001.tif]

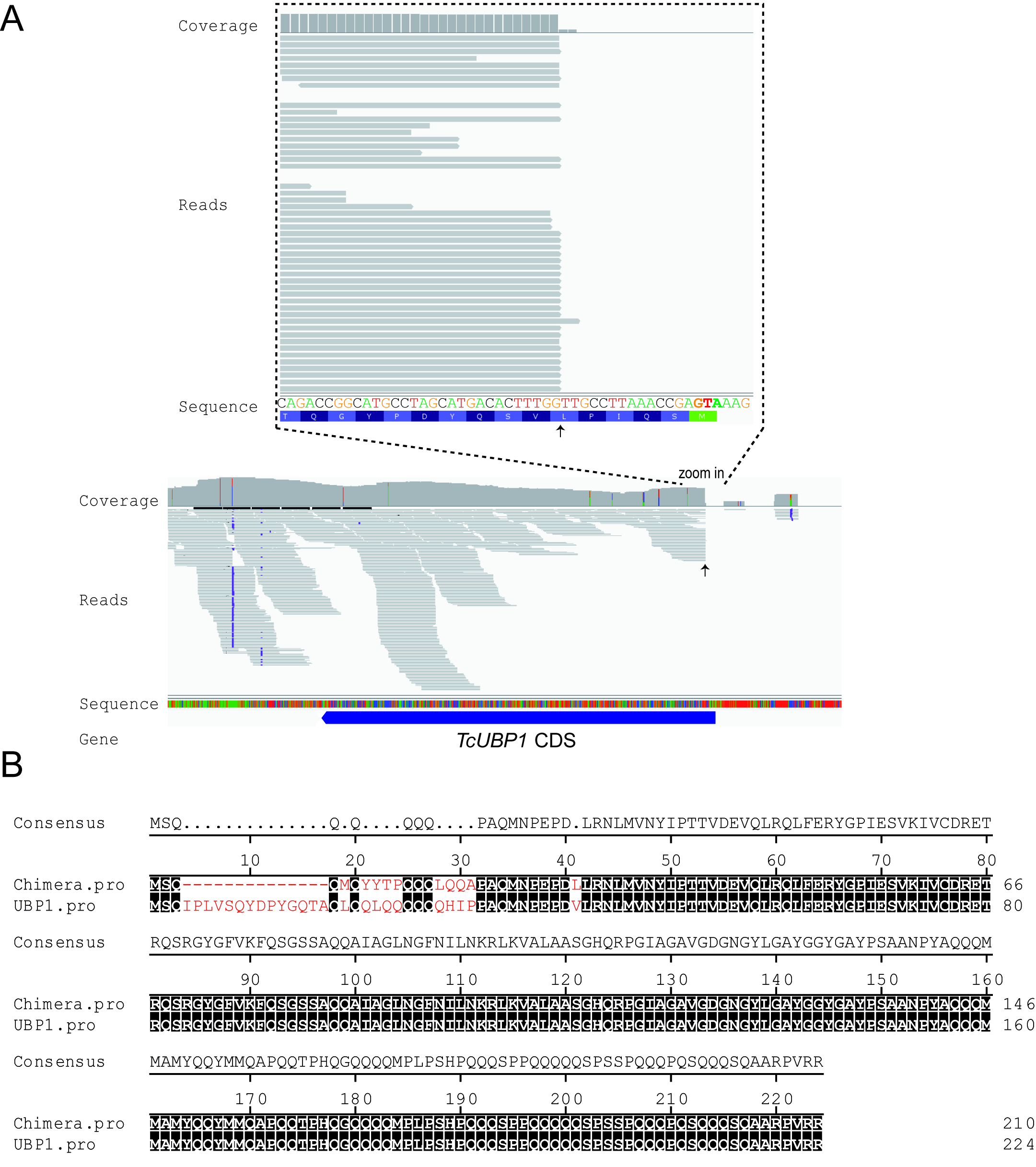

Supplement: S2 Fig — A, Mapped reads were visualized using IGV software. Coverage of reads is shown on the upper part of the IGV image. The scheme shows the 5′-end of TcUBP1 CDS and the arrow indicates the position where the hygromycin gene was inserted. B, Pairwise sequence comparison between TcUBP1 and TcUBP1mut proteins obtained with the Jotun Hein Method. Sequences were aligned using Lasergene package (DNASTAR Inc.). (TIF) [file pntd.0012179.s002.tif]

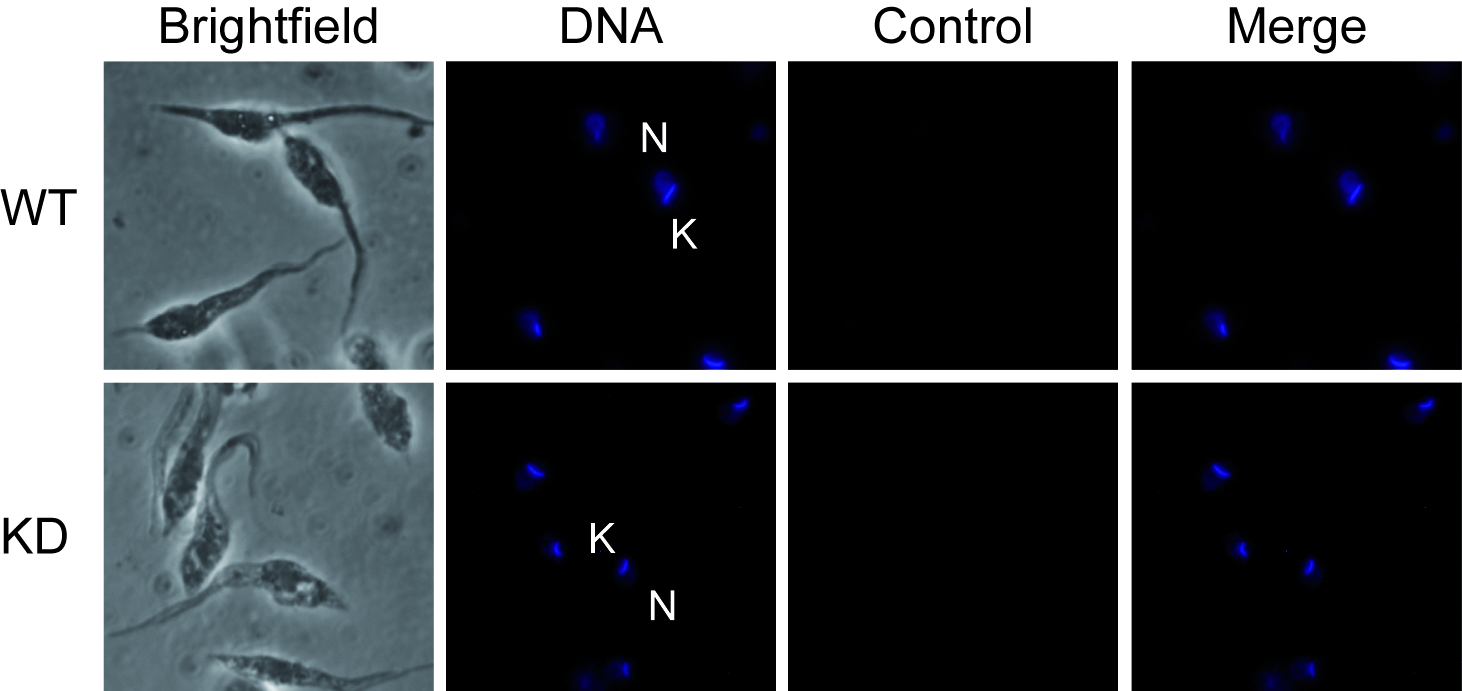

Supplement: S3 Fig — Immunohistochemical staining of WT and KD samples processed without the primary antibody (red channel, control). In the DAPI panel (blue channel, DNA), the nuclear (N) and kinetoplast (K) DNA of T. cruzi are indicated. (TIF) [file pntd.0012179.s003.tif]

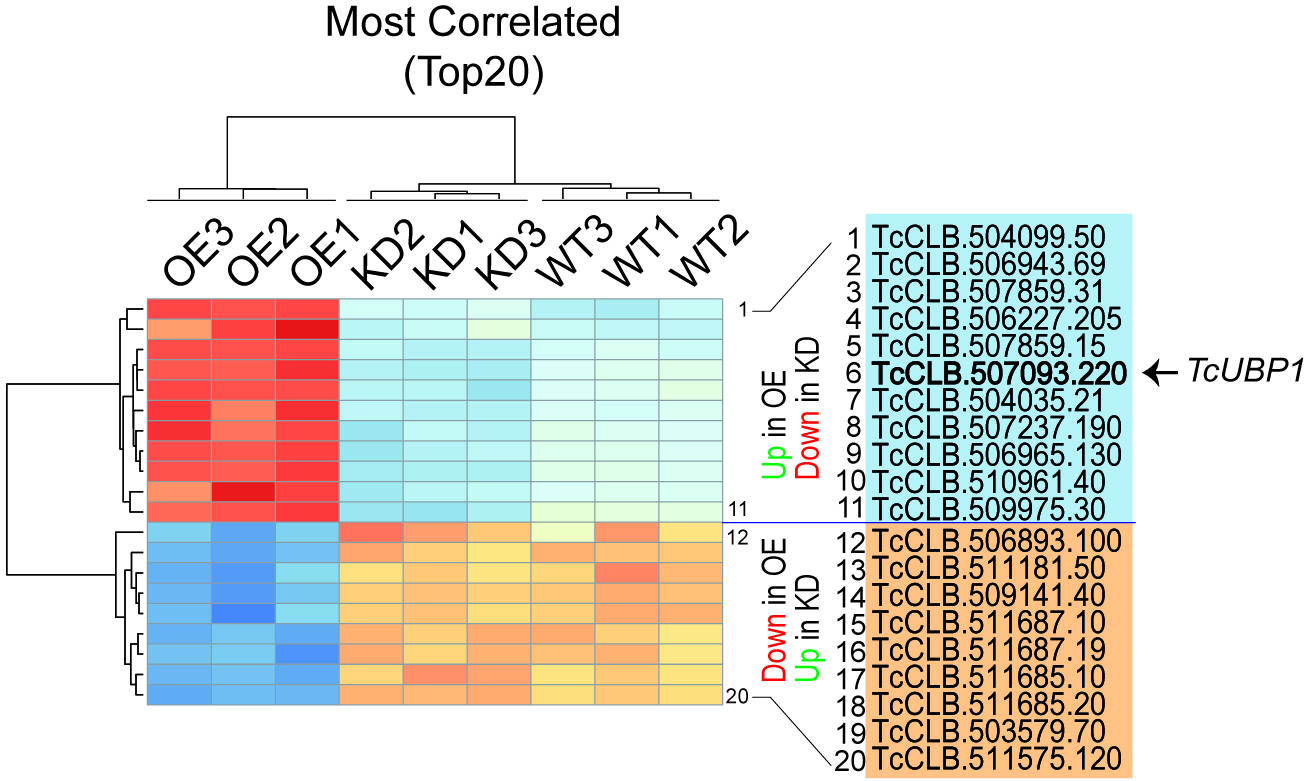

Supplement: S4 Fig — The key is as for Fig 4B. The Z-score scale bar represents relative expression +/- SD from the mean. (TIF) [file pntd.0012179.s004.tif]

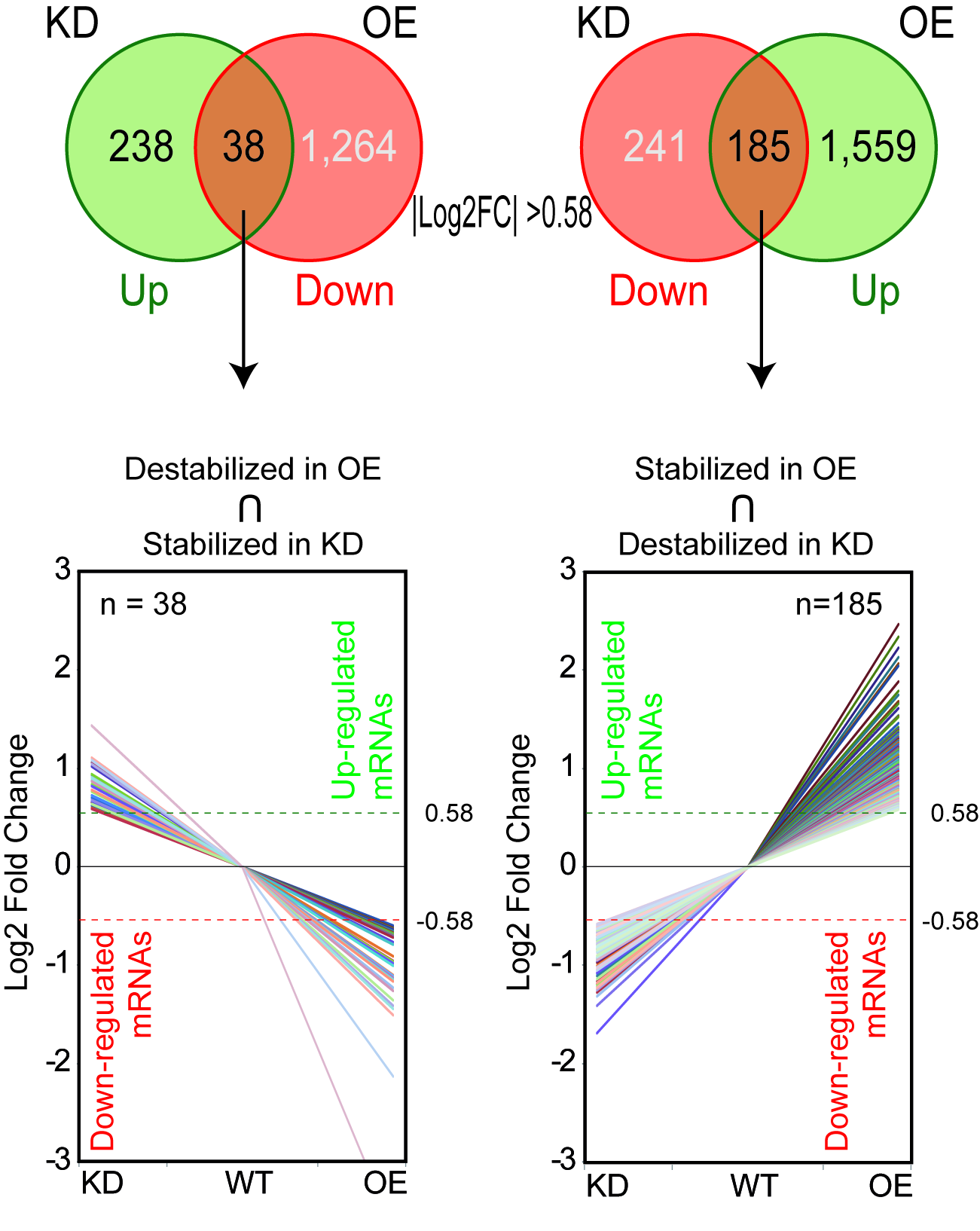

Supplement: S5 Fig — The key is as for Fig 5 but showing the number of genes 1.5-fold regulated in each condition OE and KD with respect to the WT control (|log2 fold change| >0.58). TcUBP1 (TcCLB.507093.220) is not plotted, due to its extreme scale. (TIF) [file pntd.0012179.s005.tif]

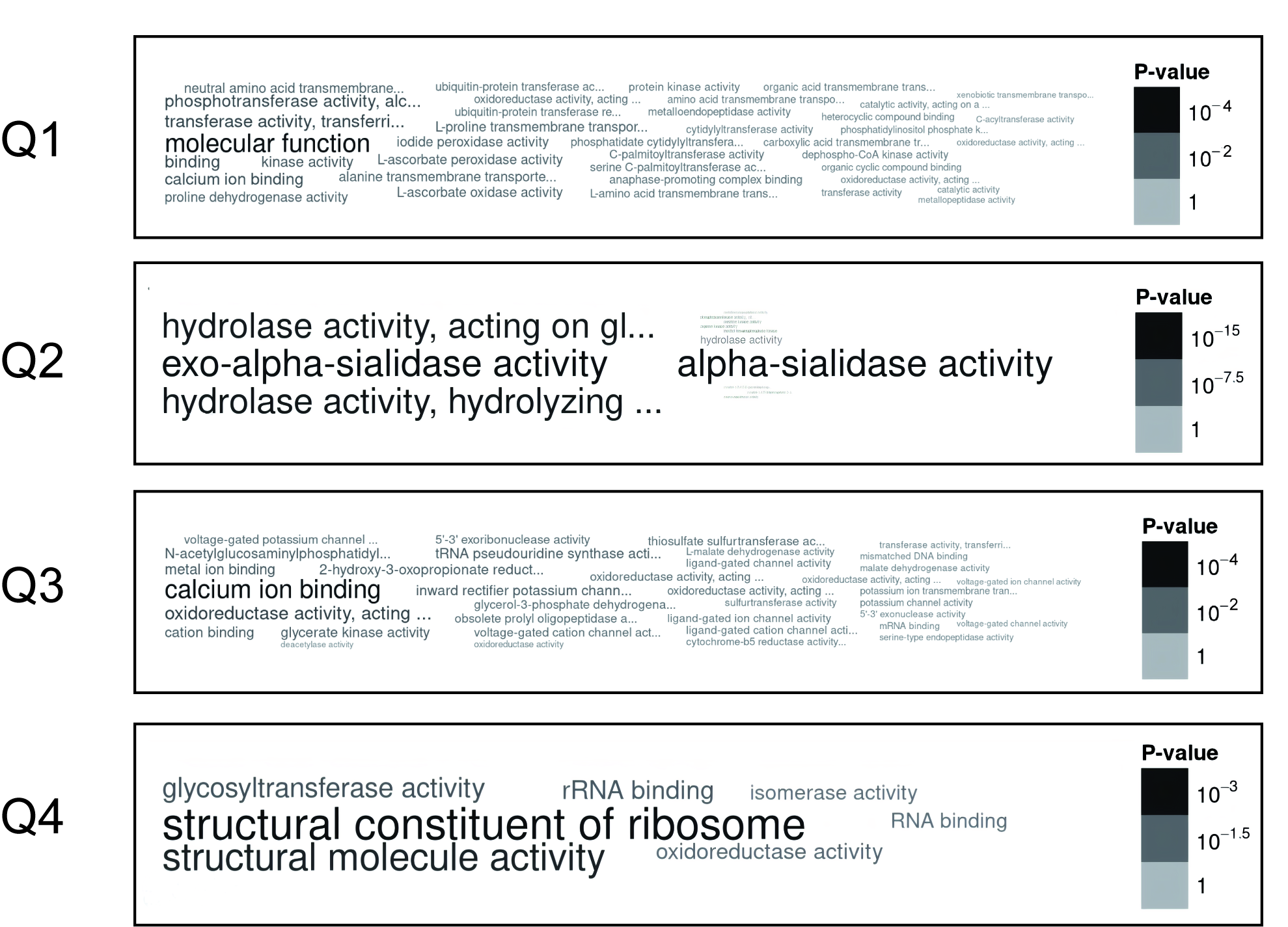

Supplement: S6 Fig — GO classification of DEGs using a criterion of at least 1.5-fold change (|log2 fold change| > 0.58); the graphs show a word cloud of terms for each quadrant of Fig 4D (only for GO domain: molecular function). Q1, n = 61 genes; Q2, n = 185 genes; Q3, n = 45 genes; Q4, n = 38 genes. (TIF) [file pntd.0012179.s006.tif]

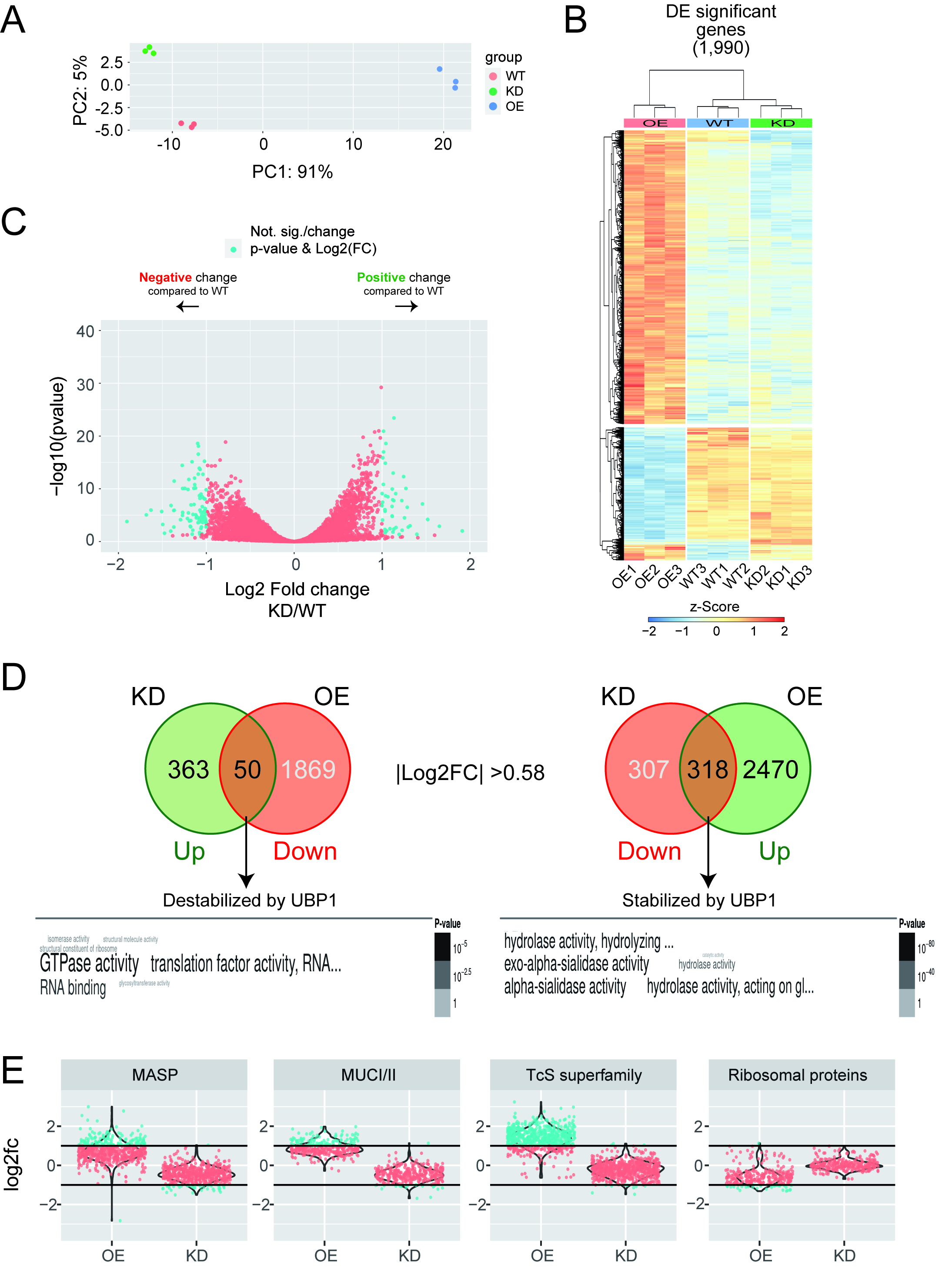

Supplement: S7 Fig — A, PCA plot displaying all 9 samples along PC1 and PC2, which describe 91% and 5% of the variability, respectively, within the expression data set. B, Heatmap and complete linkage clustering using all replicates per group, 1,990 significant genes with |log2 fold change | > 1 were clustered. The key is as for Fig 4B. C, volcano plot showing the differential expression analysis of genes in UBP1mut-KD and WT parasites. Tomato and cyan dots show nonsignificant and significant DEGs, respectively. D, Venn diagrams showing the number of genes 1.5-fold affected in each condition (OE and KD) with respect to the WT control (|log2 fold change| >0.58). The graphs show the word clouds of GO terms generated from the gene lists obtained from each intersection (only for GO domain: molecular function). E, Violin plots displaying the expression distribution of the genes within four categories in the OE or KD transcriptomes relative to the WT control (log2 fold change, FDR-adjusted p value < 10%). Categories in the figure are indicated at the top of each panel; they display significantly different values between the two conditions (Student’s t-test, p value < 0.001). (TIF) [file pntd.0012179.s007.tif]

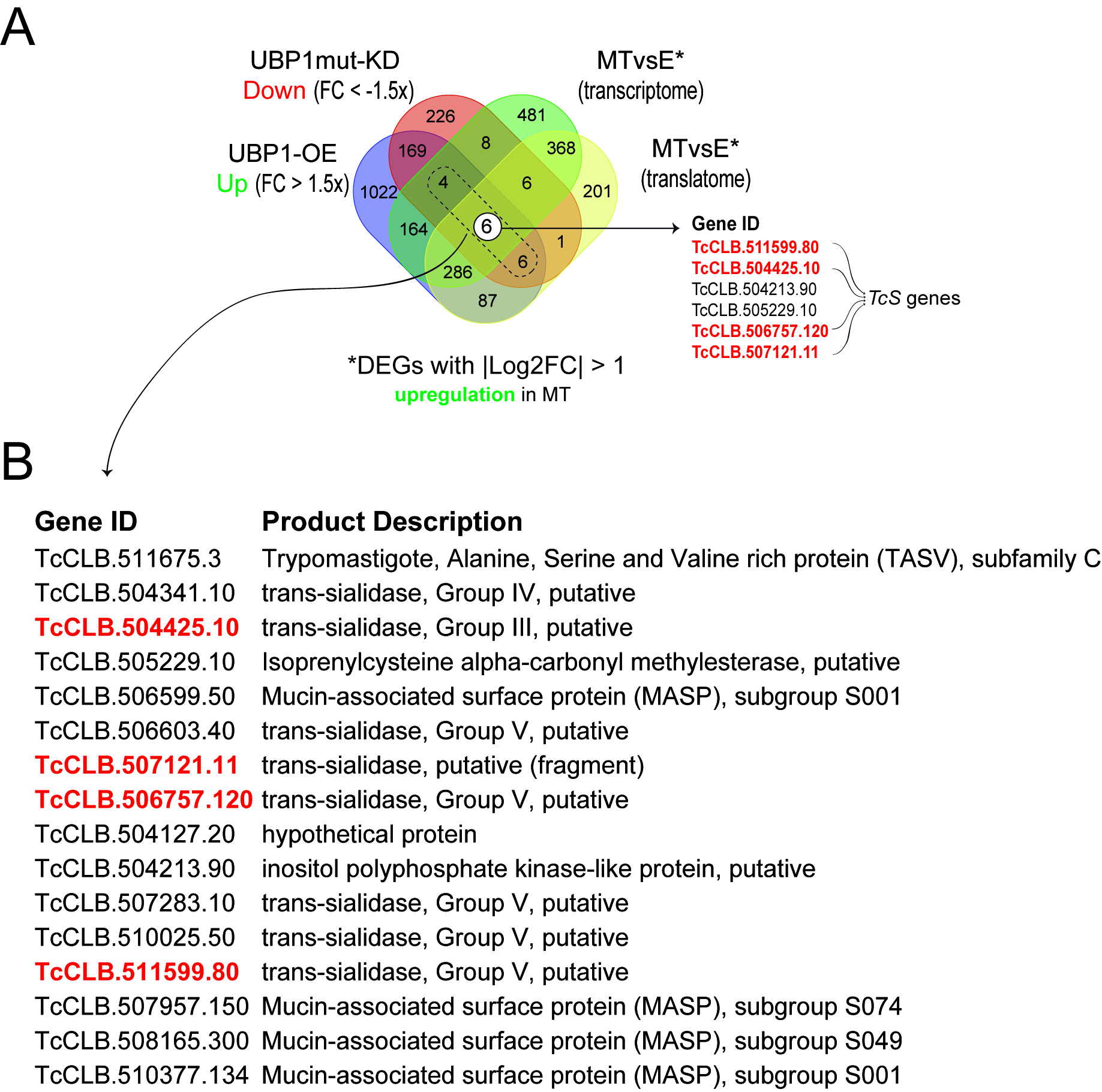

Supplement: S8 Fig — A, UBP1-OE upregulated (log2 fold change >0.58), UBP1-mutKD downregulated (log2 fold change < -0.58), and transcriptome or translatome upregulated in MT (log2 fold change >1) are shown. B, list of the intersection of genes belonging to UBP1-OE (upregulated) ∩ UBP1mut-KD (downregulated) ∩ [MTvsE-transcriptome (upregulated) ∪ MTvsE-translatome (upregulated)]. In red, glycoprotein members of the TcS superfamily. (TIF) [file pntd.0012179.s008.tif]
